# Headspace Solid-Phase Microextraction/Gas Chromatography–Mass Spectrometry for the Determination of 2-Nonenal and Its Application to Body Odor Analysis

**DOI:** 10.3390/molecules26195739

**Published:** 2021-09-22

**Authors:** Keita Saito, Yoshiyuki Tokorodani, Chihiro Sakamoto, Hiroyuki Kataoka

**Affiliations:** School of Pharmacy, Shujitsu University, Nishigawara, Okayama 703-8516, Japan; sh1908055@outlook.jp (Y.T.); sh1909027@outlook.jp (C.S.); hkataoka@shujitsu.ac.jp (H.K.)

**Keywords:** body odor, 2-nonenal, wiping method, pasting method, solid-phase microextraction (HS–SPME), gas chromatography–mass spectrometry (GC–MS)

## Abstract

The odors and emanations released from the human body can provide important information about the health status of individuals and the presence or absence of diseases. Since these components often emanate from the body surface in very small quantities, a simple sampling and sensitive analytical method is required. In this study, we developed a non-invasive analytical method for the measurement of the body odor component 2-nonenal by headspace solid-phase microextraction coupled with gas chromatography–mass spectrometry by selective ion monitoring. Using a StableFlex PDMS/DVB fiber, 2-nonenal was efficiently extracted and enriched by fiber exposition at 50 °C for 45 min and was separated within 10 min using a DB−1 capillary column. Body odor sample was easily collected by gauze wiping. The limit of detection of 2-nonenal collected in gauze was 22 pg (S/N = 3), and the linearity was obtained in the range of 1–50 ng with a correlation coefficient of 0.991. The method successfully analyzed 2-nonenal in skin emissions and secretions and was applied to the analysis of body odor changes in various lifestyles, including the use of cosmetics, food intake, cigarette smoking, and stress load.

## 1. Introduction

Body odor may be an indicator of stress and lifestyle diseases, including psychosomatic disorders, and may be an obstacle to a healthy social life [1,2,3,4]. As people are reluctant to indicate that others have body odor and the judgment criteria are ambiguous, the development of an objective quantitative method to evaluate body odor is desired. Body odor components include fatty acids, spices, and sulfurous odors [3,4]. Secretions by sweat glands and sebaceous glands are derived from volatile components decomposed by skin bacteria as well as components external to the body [3,4]. Among these compounds are unsaturated aldehydes, such as 2-nonenal, which is produced by the oxidation and decomposition of 9-hexadecenoic acid secreted by the sebaceous glands [1] (Figure S1). 2-Nonenal is also known as the odor contained in foods such as fresh juice and beer, and several analytical methods have been reported [5,6,7,8,9,10,11,12,13,14,15]. To date, however, sampling and analytical methods required to quantitatively analyze body odor compounds have not been established, so the actual state and dynamics of their occurrence have not been fully determined [3,4,16]. As “odors” generated from the body surface are complex and quantitatively small, their components may be difficult to detect with an analytical instrument. Highly sensitive analytical methods with efficient sampling and pretreatment are therefore needed to separate, identify, and quantify odor compounds. Although stir bar sorptive extraction [10], single-drop microextraction [11], and headspace solid-phase microextraction (HS–SPME) [12] methods have been reported for the efficient preconcentration of 2-nonenal in foods, they have not yet been used for body odor analysis. A conventional sampling method of skin emissions and secretions consists of placing the palm of the hand in a PVF bag and collecting the generated gas [1], but this method has drawbacks, including a possible lack of airtightness and questions about quantitative sampling. Body odor compounds have also been analyzed by gas chromatography (GC) after headspace sampling or passive flux sampling [1,17,18], but these methods have problems with cost and storage. The SPME, using a fused-silica fiber externally coated with an appropriate stationary phase, is an effective sample preparation technique for integrating several operations such as sample collection, extraction, analyte enrichment, and isolation from sample matrices and is easily coupled with GC [4,19,20,21]. It is fast, solvent-free, and cost-effective and can improve the detection limits. 

2-Nonenal is produced by reactions between fatty acids and lipid peroxides and is involved in the intake of fats and foods rich in fatty acids and in the peroxidation of lipids [1]. Thus, this production and intake of this compound are associated with the generation of active oxygen species [2]. Drinking, smoking, stress, and other lifestyle factors may therefore affect 2-nonenal secretion and alter body odor. Therefore, the effects of internal and external factors such as dietary intake, cosmetic use, smoking, and stress on body odor changes were analyzed using the method developed in this study.

In this study, we developed an efficient sampling method for the collection of body odor and an HS–SPME coupled with GC–mass spectrometry (GC–MS) for the selective and sensitive analysis of 2-nonenal in skin emissions and secretions. The method was applied to the body odor analysis in various lifestyles.

## 2. Results and Discussion

### 2.1. Detection of 2-Nonenal by Gas Chromatography–Mass Spectrometry

**Figure 1:**
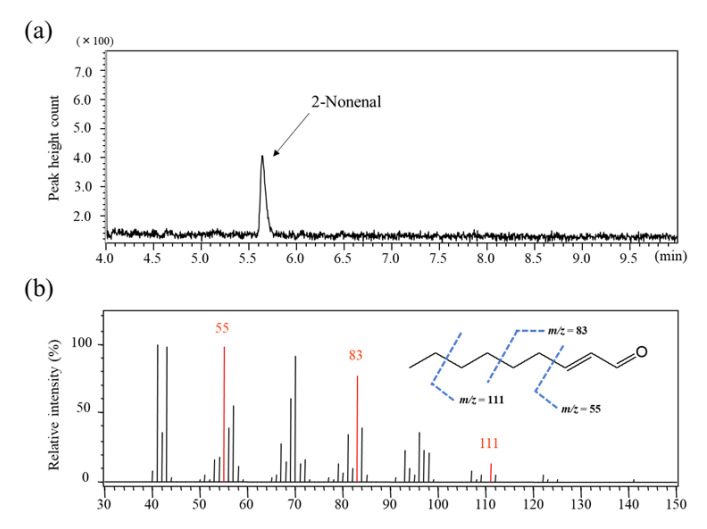
Chromatogram (**a**) and mass spectrum (**b**) obtained from standard 2-nonenal. The dotted lines in the structure represent the fragments of 2-nonenal.

Full scan mass spectrum was measured at an *m*/*z* range from 30 to 150 amu in order to determine the selected monitoring ion (SIM) for 2-nonenal (Mw: 140 daltons). Figure 1a shows a typical total ion chromatogram of 2-nonenal fragments obtained by 1 µL direct injection analysis of standard 2-nonenal (1 µg/mL solution). 2-Nonenal was eluted as a single and symmetrical peak within 10 min using a DB−1 column. As shown in Figure 1b, no molecular ions peak was detected, but several fragment ion peaks produced by cleavage of the 2-nonenal structure were observed on the spectra. Among these fragment ions, *m*/*z* = 55 [CH=CHCHO], *m*/*z* = 83 [CH_2_CH_2_CH=CHCHO], and *m*/*z* = 111 [CH_2_CH_2_CH_2_CH_2_CH=CHCHO]) were selected for SIM mode detection. 

### 2.2. Optimization of Headspace Solid-Phase Microextraction and Desorption

**Figure 2:**
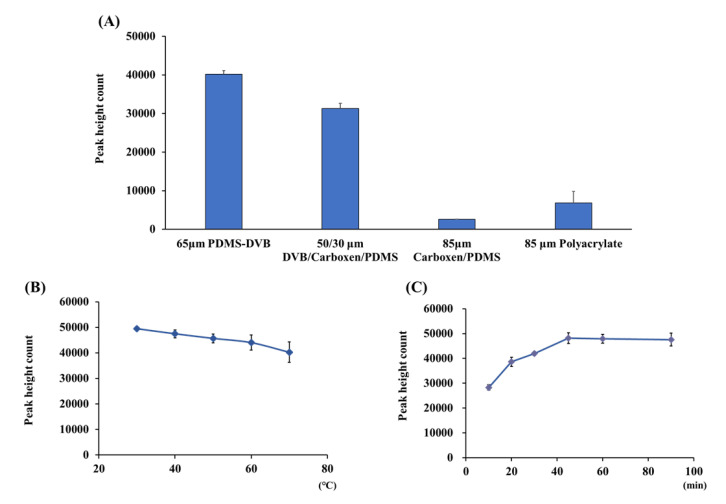
Effects of (**A**) fiber coatings, (**B**) temperature, and (**C**) time on the HS–SPME of 2-nonenal. For HS–SPME, 100 ng of 2-nonenal in gauze was extracted by (**A**) fiber exposition at 50 °C for 45 min, (**B**) for 45 min with PDMS-DVB, and (**C**) at 50 °C with PDMS-DVB.

In the SPME method, fiber coatings with high distribution coefficients must be used to increase the extraction efficiency of the compounds. To select an SPME fiber, the four different commercially available fibers (PDMS/DVB, DVB/CAR/PDMS, CAR/PDMS, and polyacrylate) were tested for extraction of 100 ng of the 2-nonenal standard solution by HS–SPME. Of these fibers, the 65 μM StableFlex PDMS/DVB fiber extracted 2-nonenal most efficiently (Figure 2A), and the fiber was used in subsequent experiments. To optimize HS–SPME extraction conditions, 100 ng of 2-nonenal standard solution in a 40 mL vial was exposed to 65 μM StableFlex PDMS/DVB fiber at various temperature and times. These experiments showed that 2-nonenal could be efficiently extracted and concentrated by exposing the fiber to the headspace at 50 °C (Figure 2B) for 45 min (Figure 2C). Although the extraction efficiency was highest at 30 °C, the extraction temperature set at 50 °C was less affected by the outside air temperature and could be kept. On the other hand, the time of the fiber exposition in the GC sample vaporization chamber at 230 °C was tested to optimize the desorption condition. The 2-nonenal extracted on the fiber was easily desorbed within 5 min by heating in the GC sample vaporization chamber, and carryover was not observed because the fiber was washed during exposition. 

**Figure 3:**
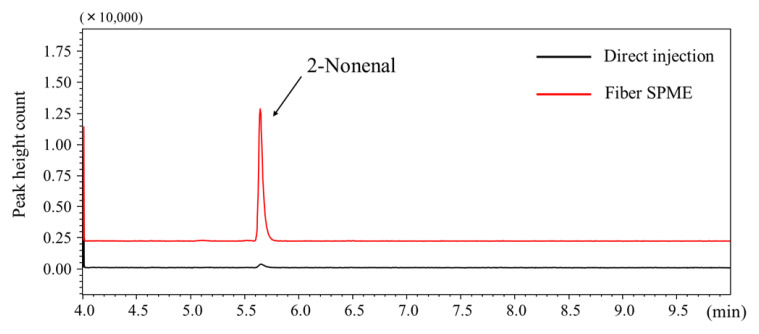
Typical total ion chromatogram of 2-nonenal obtained by (a) direct injection (200 ng/mL solution, 1 µL) and HS–SPME (200 ng/mL solution, 100 µL in 40-mL vial).

The absolute amount of 2-nonenal extracted by the HS–SPME method was calculated by comparing peak height count with direct injection onto the GC column. From the sample of 20 ng, 8.0 ng (40%) of 2-nonenal was extracted onto the PDMS/DVB fiber by HS–SPME under optimized conditions. Although the extraction yield was low, the reproducibility was good (relative standard deviation: RSD = 4.7%, *n* = 3). Figure 3 shows the chromatograms obtained by direct injection and HS–SPME techniques. The sensitivity of the SPME method showed a 40-fold increase in sensitivity compared with when a solution of the same concentration was directly injected (1 µL). 

### 2.3. Sampling of Skin Emissions and Secretions

Four sampling methods (see the Materials and Methods section) for the collection of skin emissions and secretions from the palm were examined. As shown in Table 1, the wiping method (method C), in which the palm surface is wiped with gauze, was found to be the most efficient in collecting 2-nonenal. On the other hand, 2-nonenal was collected by the solution method (method A) using water in a cup and by the pasting method (method D) using gauze taped to the palm but not by the headspace method (method B) using HS–SPME directly in a suction cup. These results indicate that 2-nonenal released from the skin surface is difficult to collect from the headspace at body temperature and that active wiping is more effective than passive sampling, even when the sampling material is in direct contact with the skin. Therefore, the wiping method was used as the sampling method for the collection of skin emissions and secretions in this study. The recovery of 2-nonenal from the gauze soaked with 5 ng of 2-nonenal was about 60% compared to that of 2-nonenal added directly to the vial without gauze.

To examine the stability of 2-nonenal collected on gauze by the wiping method, 0.1 g of gauze soaked with 100 ng of 2-nonenal was stored in vials at various times and temperatures, and the amount of 2-nonenal remaining on the gauze was determined by HS–SPME/GC–MS. As shown in Table 2, when stored at room temperature, there was almost no decrease in 2-nonenal up to 6 h, but it was found to decrease to 60% at 24 h. However, if the gauze was kept refrigerated at 4 °C, there was almost no decrease even after 24 h. Therefore, the gauze containing 2-nonenal collected by the wiping method should be kept refrigerated until analysis. 

### 2.4. Analytical Method Validation by Wiping Method

To validate a proposed method based on the wiping sample collection, standard 2-nonenal placed in a petri dish was wiped with 0.1 g of dry gauze and analyzed by HS–SPME/GC–MS. Linearity was validated by triplicate analyses for 2-nonenal at 0, 1, 2, 5, 10, 20, and 50 ng. The calibration curve was linear with a correlation coefficient of 0.991, and the relative standard deviations (RSDs) of peak height counts at each point ranged from 1.8 to 9.6 % (*n* = 3). The LOD (S/N = 3) and LOQ (S/N = 10) of 2-nonenal in gauze were 22 pg and 74 pg, respectively. Intra-day and inter-day precisions expressed as RSD (%) were found to be 9.6 and 3.6%, respectively. These results show that the method is highly sensitive and reproducible for the determination of 2-nonenal by the wiping method.

### 2.5. Analyses of 2-Nonenal in the Body Odor Collected from Several Body Parts

**Figure 4:**
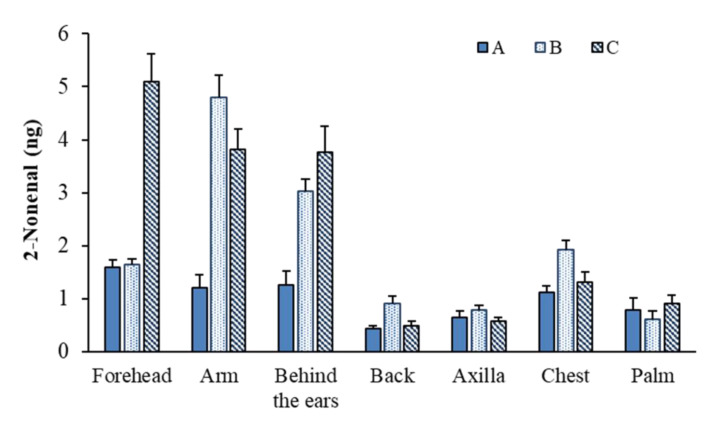
Comparison of 2-nonenal contents in body odor samples collected from several body parts (A: female in their 20 s, B: male in their 20 s, C: male in their 30 s).

**Figure 5:**
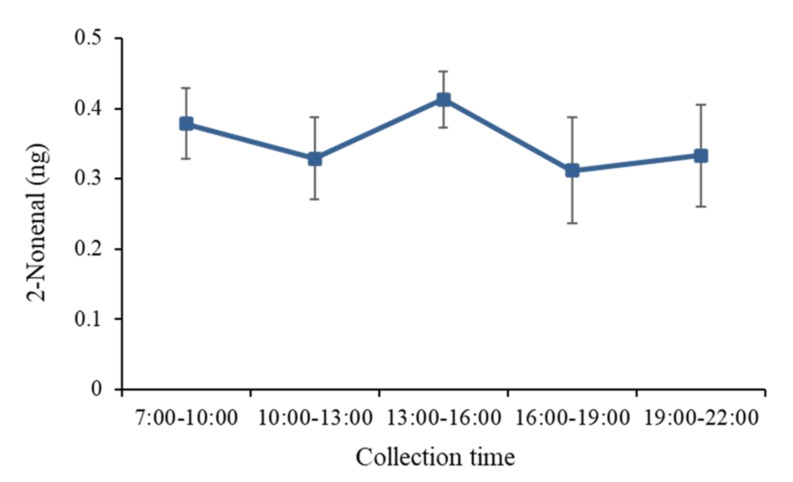
Circadian rhythm of 2-nonenal emission from skin (forehead) (average contents of 2-nonenal from three people).

**Figure 6:**
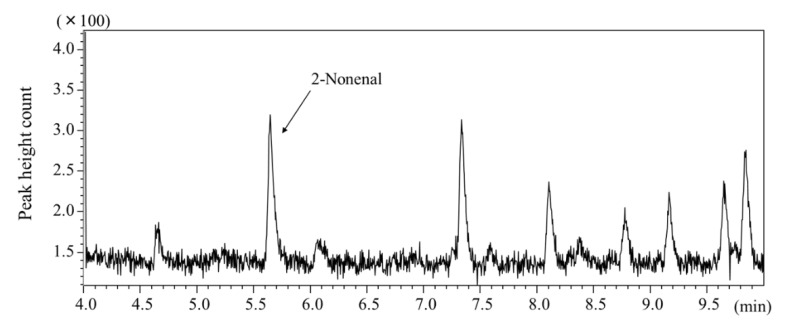
Total ion chromatogram of skin emission sample (forehead, male in their 30 s).

Body odor samples were collected by the wiping method from several body parts 6 h after washing with soap and then analyzed by HS–SPME/GC–MS. Figure 4 shows the 2-nonenal content in body odor samples collected from the arms, axillae, back, chest, forehead, palm, and behind the ears of three subjects. Relatively high amounts of 2-nonenal were detected in the samples from the foreheads, arms, and ears, although this varied between individuals. To determine the amount of 2-nonenal secreted at different times of the day, body odor samples were collected from the forehead every 3 h by the wiping method and analyzed by HS–SPME/GC–MS. As shown in Figure 5, the secretion of 2-nonenal was highest between 13:00 and 16:00 in the afternoon (average contents of 2-nonenal from three people). The chromatogram of the 2-nonenal sampled from the forehead is shown in Figure 6. It was found that 2-nonenal was detected with clear separation and was not affected by the interfering peak.

### 2.6. Application to the Analysis of Body Odor Changes in Various Lifestyles

Table 3 shows the results of HS–SPME/GC–MS analysis of the samples collected from the forehead by the wiping method after a certain period of time after each treatment. 2-Nonenal secretion was significantly increased on the high-fat diet compared with the low-fat diet. 2-Nonenal secretion was also slightly increased in lotion use and smoking compared with non-use and non-smoking, but the difference was not statistically significant. In contrast, mental stress caused by long meetings significantly increased 2-nonenal secretion. This may be because stress-induced sweating increased the secretion of fatty acids and lipid peroxides from the sebaceous glands, increasing oxidative degradation of these compounds to 2-nonenal by indigenous bacteria on the body surface [1]. 

## 3. Materials and Methods

### 3.1. Reagents and Materials

2-Nonenal was purchased from Nacalai Tesque (Kyoto, Japan). Methanol and distilled water (LC–MS grade) were purchased from Kanto Chemical (Tokyo, Japan). A stock solution of 1 mg mL^−1^ of 2-nonenal was prepared by dissolving in methanol (LC–MS grade), tightly sealed, and stored at 4 °C. This stock solution was diluted with distilled water (LC–MS grade) to the desired concentration prior to use. All other chemicals were of analytical grade.

SPME assemblies with a replaceable and reusable extraction fiber coated with StableFlexTM polydimethylsiloxane/divinylbenzene (PDMS/DVB, 65 μM), StableFlexTM carboxene/PDMS/DVB (DVB/CAR/PDMS, 50/30 μM), (CAR/ PDMS, 85 μM), StableFlexTM CAR/PDMS (85 μM), and polyacrylate (85 μM) were purchased from Supelco (Supelco Japan, Tokyo). These fibers were conditioned in a GC injection port at adequate temperature prior to use. One fiber can be used repeatedly at least more than one hundred times.

### 3.2. Gas Chromatography–Mass Spectrometry

GC–MS analysis was performed in the scan and SIM modes on a Shimadzu QP–2010 (Kyoto, Japan)gas chromatograph–mass spectrometer in conjunction with a GCMS solution Ver.2 workstation. GC separation was performed using a fused-silica capillary column of cross-linked DB−1 (J&W, Folsom, CA, USA: 60 m × 0.25 mm i.d., 1.0 μM film thickness) under the following operating conditions: injection and detective temperatures, 230 °C; column temperature, held at a temperature of 190 °C for 2 min and increased to 230 °C at a rate of 5 °C min^−1^; inlet helium carrier gas flow rate, 1 mL min^−1^ maintained by an electronic pressure controller; and split ratio, 10:1. The electron impact (EI)–MS conditions were as follows: ion-source temperature, 230 °C; ionizing voltage, 70 eV. The full scan mass spectra were obtained at an *m*/*z* range from 40 to 150 amu. Selected ion monitoring (SIM) mode detection for 2-nonenal was selected at *m*/*z* = 55, 83, 111. The peak height count was measured to construct calibration curve and to determine concentrations of 2-nonenal in samples.

### 3.3. Headspace Solid-Phase Microextraction

The sample was placed in a 40-mL screw-cap vial with a PTFE septum and heated on a hot plate at 50 °C. The SPME needle was passed through the septum of the vial, and the fiber was exposed in the headspace (HS) above sample for 45 min to adsorb the compounds vaporized by heating. After extraction, the fiber was retracted into the needle, the needle was withdrawn and introduced directly into the GC–MS sample vaporization chamber to desorb the extracted compound by heating. Then, the fiber was retracted into the needle, the needle was removed from the sample vaporization chamber and used for the HS–SPME of the next sample. An outline of the procedure for extraction by HS–SPME and desorption for GC–MS analysis is shown in Figure S2.

### 3.4. Sampling of Skin Emissions and Secretions

**Figure 7:**
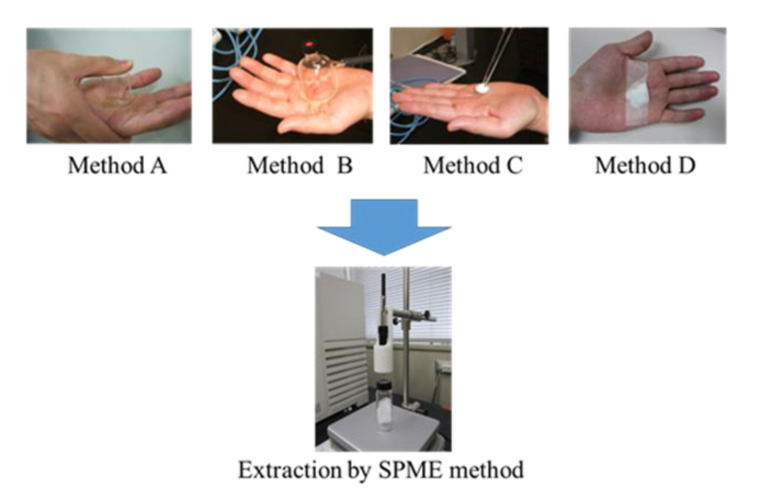
Sampling methods of skin emissions and secretions.

Four sampling methods for the collection of skin emissions and secretions were compared. Figure 7 shows the sampling methods from the palm of the hand. Sampling was carried out 3 h after washing the skin surface with soap. In method A (solution method), 2 mL of distilled water were added to a glass cup, and the cup was inverted so that the water touched the skin on the palm of one hand for 5 min. The aqueous solution was subjected to the above HS–SPME method. In method B (headspace method), a suction cup is placed on the palm, the SPME fiber needle is inserted through the septum into the cup under reduced pressure, and then, the fiber is exposed for 5 min to extract the skin emissions. In method C (wiping method), a 2 cm square area of the palm was wiped for 1 min with 0.1 g of dry gauze as a collecting material of skin emissions and secretions, which was then placed in a vial and subjected to the above HS–SPME method. In method D (pasting method), 0.1 g of dry gauze was taped to the palm and extracted for 3 h. These collecting materials were then subjected to the HS–SPME as in method C. 

### 3.5. Analysis of Body Odor Samples

The experimental protocol was approved by the Research Ethics and Safety Committee of Shujitsu University, and body odor samples were provided from one female and two male subjects in their 20 s and 30 s by informed consent. The samples were collected from the arm, axilla, back, chest, forehead, palm, and behind the ears by method C described in the above section. 2-Nonenal in the body odor collected on the gauze was subsequently analyzed by HS–SPME/GC–MS. To analyze changes in body odor due to the various life styles, body odor samples were collected from the forehead by method C before and after treatments and analyzed by HS–SPME/GC–MS as described above. As a concrete examination, we compared before and after food intake (eating 200 g of vegetables for the low fat diet or 200 g of beef for the high fat diet, sampling 12 h later), using cosmetics (applying 1 mL lotion to face, sampling 6 h later), smoking (smoking one cigarette, sampling 2 h later), and a 3 h meeting as a stress load (sampling 6 h later).

## 4. Conclusions

The method developed in this study can easily collect 2-nonenal, a component of human body odor, from the skin surface by gauze wiping and can be selectively and sensitively analyzed by HS–SPME/GC–MS. Gauze wiping is a rapid and non-invasive sampling method for skin emissions and secretions and has better storage stability than other tested methods. As shown in Table 4, the proposed method is more sensitive than previously reported methods, even when converted into the skin surface area and the amount of recovery per hour. We believe that this method is a useful tool for 2-nonenal analysis in body odor. Although this study focused on 2-nonenal, body odor is a complex mixture of compounds, and therefore, it is necessary to analyze not only 2-nonenal but also a wide range of related compounds in the future. The method developed in this study may be applicable to their comprehensive analysis. 

## Figures and Tables

**Table 1:** Comparison of skin emission sampling methods.

Classification	Sampling Method	2-Nonenal Content (ng) Mean ± SD (*n* = 3)
A	Glass cup sampling	0.47 ± 0.51
B	Direct SPME sampling	N.D. ^1^
C	Wiping method	0.90 ± 0.13
D	Pasting method	0.18 ± 0.23

^1^ N.D.: not detectable.

**Table 2:** Comparison of storage conditions after sampling by the wiping method.

Storage Condition ^1^	Storage Time (h)	Peak Height Count Mean ± SD (*n* = 3)
Room temperature	0	25,694 ± 1486
	1	25,025 ± 2830
	2	24,537 ± 1454
	3	25,968 ± 2162
	6	24,208 ± 3058
	24	15,250 ± 1600
4 °C (refrigerator)	24	24,594 ± 3679

^1^ The gauze soaked with 50 ng of 2-nonenal was stored in vials.

**Table 3:** 2-Nonenal contents detected in body odor samples in various life styles.

Treatment	Sampling Time after Treatment	2-Nonenal Content (ng) Mean ± SD (*n* = 3)	T-Test *p* Value
Low fat diet	12	0.49 ± 0.09	*p* < 0.05
High fat diet	12	0.70 ± 0.09	
Without using cosmetics	6	0.23 ± 0.04	*p* > 0.05
Using cosmetics	6	0.26 ± 0.08	
Before smoking	2	0.27 ± 0.08	*p* > 0.05
After smoking	2	0.37 ± 0.09	
Before meeting	6	0.23 ± 0.04	*p* < 0.05
After meeting	6	0.35 ± 0.09	

**Table 4:** Comparison of analytical methods for 2-nonenal and other aldehydes in body odor.

Compounds	Analytical Method	Sampling and Preconcentration	Detection Amount	Reference
2-Nonenal	GC–MS	Tedlar bag and TENAX-TA column	15.1 ± 20.4 ng·cm^−3 (1)^	[1]
Five aldehydes and acetone	HPLC–UV	Passive flux sampler (trapping filter: DNPH impregnated filter)	17 ng·cm^−2^·h^−1 (2)^	[17]
2-Nonenal and diacetyl	GC–MS	Passive flux sampler (trapping media: Monotrap, DCC18)	0.020 to 5.8 ng·cm^−2^·h^−1 (3)^	[18]
2-Nonenal	GC–MS	Fiber SPME (Wiping method)	2.4 pg·cm^−2^·h^−1 (4)^	This study

^(1)^ Sampling from the back of the shirt worn for 3 days; ^(2)^ Sampling from the surface of human skin, sampling time 1 h, aldehydes (LOD); ^(3)^ Sampling from the nape of the neck, sampling time 7 h; ^(4)^ LOD.

## Data Availability

The data presented in this study are available from the corresponding author upon request.

## Supplementary Materials

The following are available online. Figure S1: Presumed formation mechanism of 2-nonenal by lipid peroxidation. Data from Haze et al., 2001 [1], Figure S2: Procedure for extraction by headspace fiber SPME and desorption for GC–MS analysis. Data from Kataoka and Saito, 2011 [20].

## Sample Availability

Samples of the compounds are not available from the authors.
