# Supplementary material for: Headspace Solid-Phase Microextraction/Gas Chromatography–Mass Spectrometry for the Determination of 2-Nonenal and Its Application to Body Odor Analysis
Source: Molecules. 2021 Sep 22;26(19):5739. doi: 10.3390/molecules26195739 (PMC8510471; doi:10.3390/molecules26195739)
Supplement: Supplementary file 1 [file molecules-26-05739-s001.zip › molecules-1386336-supplementary.pdf]

Supplementary Materials

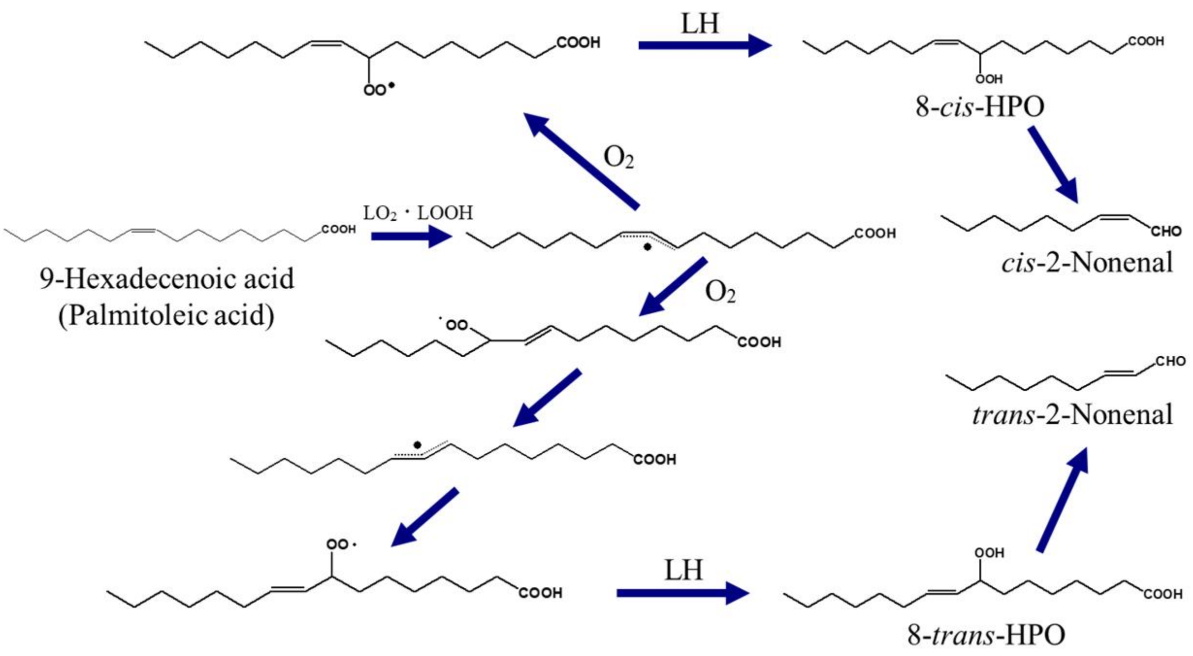

Figure S1. Presumed formation mechanism of 2-nonenal by lipid peroxidation. Data from Haze et al., 2001 [1].

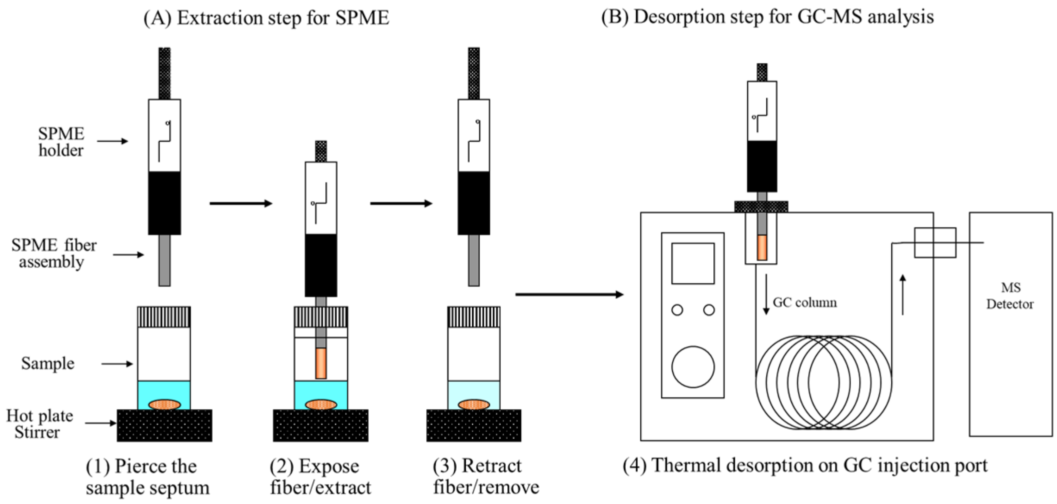

Figure S2. Procedure for extraction by headspace fiber SPME and desorption for GC–MS analysis. Data from Kataoka and Saito, 2011 [20].
